# Castor oil: a suitable green source of capping agent for nanoparticle syntheses and facile surface functionalization

**DOI:** 10.1098/rsos.180824

**Published:** 2018-08-15

**Authors:** M. B. Mensah, J. A. M. Awudza, P. O'Brien

**Affiliations:** 1 Department of Chemistry, Kwame Nkrumah University of Science and Technology, PMB, Kumasi, Ghana; 2 School of Chemistry and School of Materials, The University of Manchester, Oxford Road, Manchester M13 9PL, UK

**Keywords:** nanoparticles, vegetable oil, castor oil, ricinoleic acid

## Abstract

Castor oil (CO) is an inedible vegetable oil (VO) that has been employed extensively as a bioresource material for the synthesis of biodegradable polymers, cosmetics, lubricants, biofuels, coatings and adhesives. It is used in medicine, pharmaceuticals and biorefineries, due to its versatile chemistry. However, there has been less focus on CO as an alternative to toxic and expensive solvents, and capping/stabilizing agents routinely used in nanoparticle syntheses. It provides a richer chemistry than edible VOs as a solvent for green syntheses of nanoparticles. CO, being the only rich source of ricinoleic acid (RA), has been used as a solvent, co-solvent, stabilizing agent and polyol for the formation of polymer–nanoparticle composites. RA is a suitable alternative to oleic acid used as a capping and/or stabilizing agent. Unlike oleic acid, it provides a facile route to the functionalization of surfaces of nanoparticles and the coating of nanoparticles with polymers. For applications requiring more polar organic solvents, RA is more preferred than oleic acid. In this review, we discuss the production, chemical and physical properties, triglyceride and fatty acid (FA) compositions and applications of CO, focusing on the use of CO and RA as well as other VOs and FAs in syntheses of nanoparticles and surface functionalization.

## 1. Introduction

The impact of nanotechnology on society is enormous. However, the use of expensive and toxic materials for the syntheses of nanoparticles is becoming a critical concern. Many researchers have resorted to employing environmentally friendly renewable bioresource materials such as vegetable oils (VOs), carbohydrates and plant extracts in the syntheses of nanoparticles [1–15]. Castor oil (CO) has been viewed as one of the most likely sources of plant oils suitable as solvents and/or capping agents for syntheses of metal and metal chalcogenide nanoparticles [14,16].

**Figure 1.**
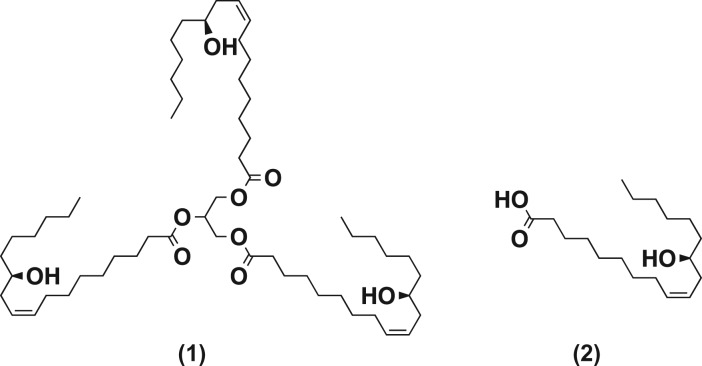
Chemical structures of triricinolein (1) and ricinoleic acid (2).

*Oleum Palmae Christi* (or CO) is a hydroxylated lipid obtained from the seed of the castor plant, *Ricinus communis* L. of the family Euphorbiaceae and native to tropical Asia and Africa [16–18]. It is considered a very important bioresource material for a wide variety of applications [19]. The triglycerides of CO consist of fatty acids (FAs), of which approximately 90% is ricinoleic acid (RA), a hydroxylated monounsaturated 18-carbon carboxylic acid (figure 1). It is the only pure source of RA [2,20,21].

RA is a multifunctional compound, possessing a carboxylic acid, a double bond (between C9 and C10) and a secondary alcohol or hydroxyl (at C12) functional group. The hydroxyl group is beta to the double bond and protects that double bond from peroxide formation [22]. These functional groups make CO (and its derivatives) completely soluble in all alcohols and present viscosities that are up to approximately sevenfold higher than the viscosities of other VOs [23]. The triricinolein (in CO, figure 1) undergoes amidation, saponification, reduction, esterification, alcoholysis, hydrolysis, dehydration, caustic fusion, sulfonation, pyrolysis, oxidation, polymerization, halogenation, epoxidation, hydrogenation and olefin metathesis reactions [18,19,24,25]. Thus, CO is applicable as a valuable raw material in industries for production of coatings, biopolymers, paints, adhesives, cosmetics, lubricants, hydraulic fluids, inks, linoleum and chemicals including sebacic acid and undecylenic acid used in the production of plasticizers and nylon [23–26]. The properties of CO that make it appropriate as a solvent for nanoparticle syntheses are: (i) non-toxicity, (ii) high boiling temperature of 313°C, (iii) colourless to pale yellow liquid, (iv) mild or no odour or taste and (v) a clear liquid at room temperature and showing no solid fat at 0°C [27,28]. The alkyl groups impose a steric effect that controls the growth, crystal structure, morphology and surface characteristics of the nanoparticles [29,30].

Carboxylic acids such as oleic acid and stearic acid have been employed extensively as ligands or capping agents [31,32]. RA also represents a valuable alternative capping agent. The presence of the hydroxyl group attached to RA-capped nanoparticles allows them to be functionalized easily with other groups to enhance their dispersion in different solvent media specific to a particular application. However, the capping activities of CO and RA have not been extensively explored. CO has been reviewed as a vital bioresource of industrial raw material for production of different functional materials, and a majority of the reviews on it are centred on developing biodegradable polymers [19,33,34]. It remains as the most promising source of building blocks for the synthesis of polyurethane and polyesters [25,35–37]. CO has also been reviewed as a source of biobased chemicals and biodiesel [16,38,39]. Although CO (and RA) has a probable significant impact on nanoparticle syntheses and applications, it has been less emphasized, and there is little information regarding its innovative development in nanotechnology. Thus, this review examines CO (and RA) as valuable bioresource material for nanoparticle syntheses and functionalization.

This review is split into four main sections: (i) facts about CO (brief history on castor cultivation, production and physico-chemical properties), (ii) composition and structure of CO and isolation of RA, (iii) application of CO in biomedicine, biopolymers, biochemicals, bioenergy, lubricants and coatings and (iv) utilization of CO (as well as other VOs) and RA (as well as other FAs) as capping ligands or solvents for nanoparticle syntheses and functionalization. The review is then concluded by highlighting the areas in nanoparticle syntheses where CO and RA can be used.

## 2. Facts about castor oil

### 2.1. Cultivation of castor oil seed

**Figure 2.**
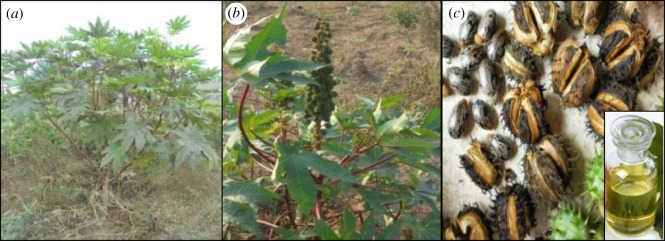
Castor plant: (*a*) matured castor plant, (*b*) bunch of castor seeds and (*c*) dried castor seed pod, seed and oil.

Ethiopia (east Africa) is believed to be the most likely origin of castor in addition to places such as northwest and southwest Asia, the Arabian Peninsula and the subcontinent of India and China [40]. The history of the castor plant cultivation is well reported by Anjani [40]. The castor plant is cultivated for its seed oil, which is the only commercial source of RA for the chemical and pharmaceutical industries [41]. The CO plant is also used as an ornamental and in insect traps [40,42]. The castor plant is a cross-pollinated diploid (*2n* *=* *2x* *=* 20) species within the family of Euphorbiaceae and the genus *Ricinus*. The castor plant is a coarse perennial crop that grows to approximately 10 ft in the tropics and has a stem diameter of 7.5–15 cm. In the temperate regions, the castor plant behaves as an annual crop with succulent stems and usually herbaceous [43]. Pictures of the castor plant, seeds and oil are shown in figure 2. CO possesses nauseating properties, and the seed kernel contains a poisonous glycoprotein called ricin. Nonetheless, the oil itself is not toxic, and the seed cake is usually detoxified using Ca(OH)_2_, NaOH or NaOCl to remove the toxins [16,38,44]. Detailed information about castor plant cultivation and CO extraction can be found in www.castoroil.in [43].

### 2.2. Production of castor oil seed

**Figure 3.**
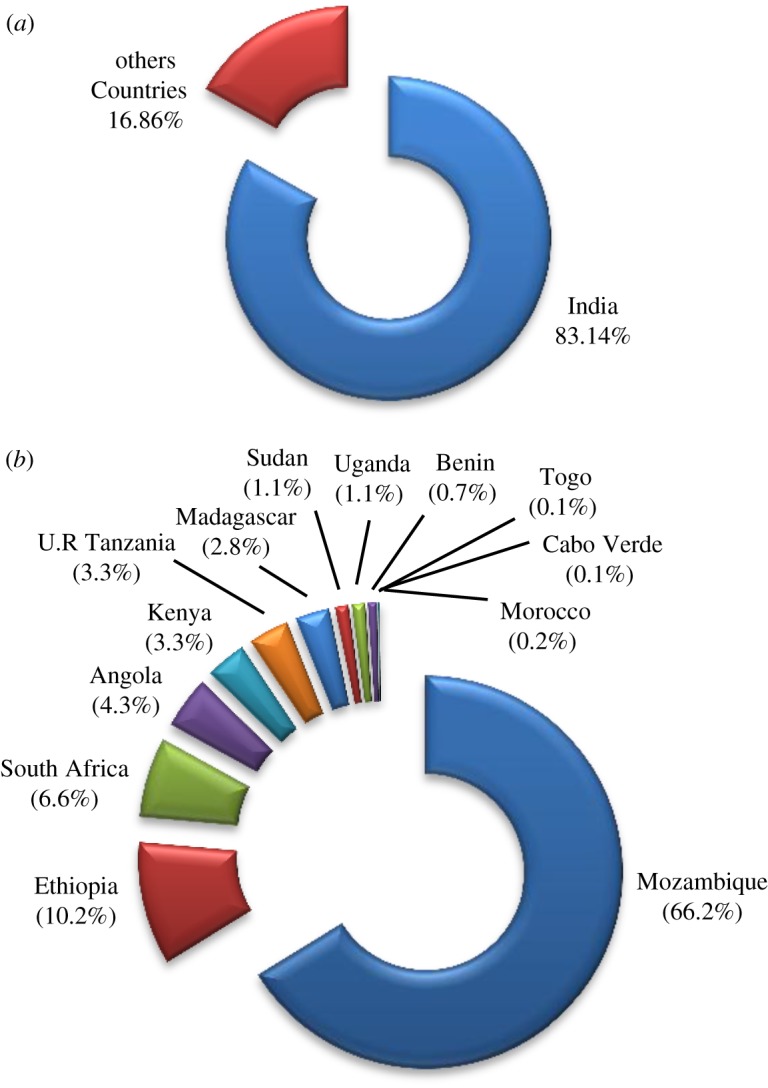
Charts showing production of CO seeds: (*a*) in the world and (*b*) in Africa, considering production figures from 2009 to 2013 obtained from FAOSTAT [46].

The oil content of the castor seed is approximately 45–50%, with a yield of 470 kg of oil per hectare [28,45]. The average annual world production of CO seed from 2009 to 2013 was approximately 1.99 × 10^6^ t [46]. The major producers of CO seed in Africa and other parts of the world are presented in table 1 and figure 3. India is the leading producer of castor seed oil. India produces approximately 83% of the world's annual production. Africa produces 90.3 × 10^3^ t of castor seeds annually, representing approximately 4.54% of the world's production. Mozambique produces approximately 3.01% of the world's annual production and is the leading producer of CO seed in Africa. Ethiopia, South Africa, Angola, Tanzania and Kenya are African countries also involved in castor production though their production figures are low. China, Brazil, Paraguay and Thailand are also noted for castor production [46].

**Table 1.** Major producers of CO seed (10^3^ t) in the world (FAOSTAT [46]).

country	2009	2010	2011	2012	2013
India	1009.0	1350.0	2295.0	1964.0	1644.0
China	170.0	150.0	180.0	120.0	60.0
Brazil	91.1	95.2	120.2	26.0	12.0
Mozambique	57.0	60.0	60.0	62.0	60.0
Paraguay	13.0	6.0	6.0	10.0	11.0
Thailand	11.7	12.2	11.3	11.0	12.0
Ethiopia	7.0	7.0	8.0	11.0	13.0
Vietnam	6.0	6.0	6.0	6.0	6.0
South Africa	5.6	6.2	5.7	6.0	6.2
Angola	3.4	4.0	4.0	4.0	4.0
World	1395.7	1720.6	2721.5	2244.9	1854.8

### 2.3. Physico-chemical properties of castor oil

The presence of the hydroxyl group on RA has a drastic effect on the viscosity, pour point, melting point, heat of fusion, solubility, crystal structure and polymorphism of CO [47]. The hydroxyl functionality induces the formation of hydrogen bonding between the hydroxylated triglyceride molecules that increases the steric hindrance of the oil, leading to the formation of weakly bound dimers and trimers of the original triglycerides; hence, CO has a high viscosity of 260.4 cSt at 40°C [48]. Molecular weights (MWs) of oils have an effect on oil viscosity. Da Silva *et al*. [49] analysed the MW of CO by vapour pressure osmometry and gas chromatography and found it to be 927.88 g mol^−1^ and 928.31 g mol^−1^, respectively. Salimon *et al*. [27] also reported the average MW of CO as 937.7 g mol^−1^. Compared with olive oil, CO has a higher MW and thus has a higher viscosity. The density of CO is also reported to be 961 kg m^−3^ [28]. CO is known as a non-drying oil because it has only one double bond (low iodine value) in each FA chain and does not harden when exposed to air [16,43]. The physico-chemical properties of CO reported by different research groups are presented in table 2.

**Table 2.** Physico-chemical properties of CO.

parameters	units	values	references
oil content	%	43.3–56.2	[27,50,51]
density	kg m^−3^	946–961	[28,50,52]
moisture content	%	0.2–3.9	[27,50,52]
iodine value	mg g^−1^	84.5–85.5	[27,53]
acid value	mg KOH g^−1^	0.03–4.9	[27,50–52]
free FA	%	0.06–3.4	[27,50,53]
hydroxyl value	mg KOH g^−1^	164.5	[52]
peroxide value	meq kg^−1^	10.2	[27]
saponification value	mg g^−1^	182.9	[27]
unsaponifiable matter		3.4	[27]
kinematic viscosity at 40°C	cSt	260.4	[48]
refractive index	—	1.47	[27]

## 3. Chemical composition and structure of castor oil

### 3.1. Triglycerides

**Figure 4.**
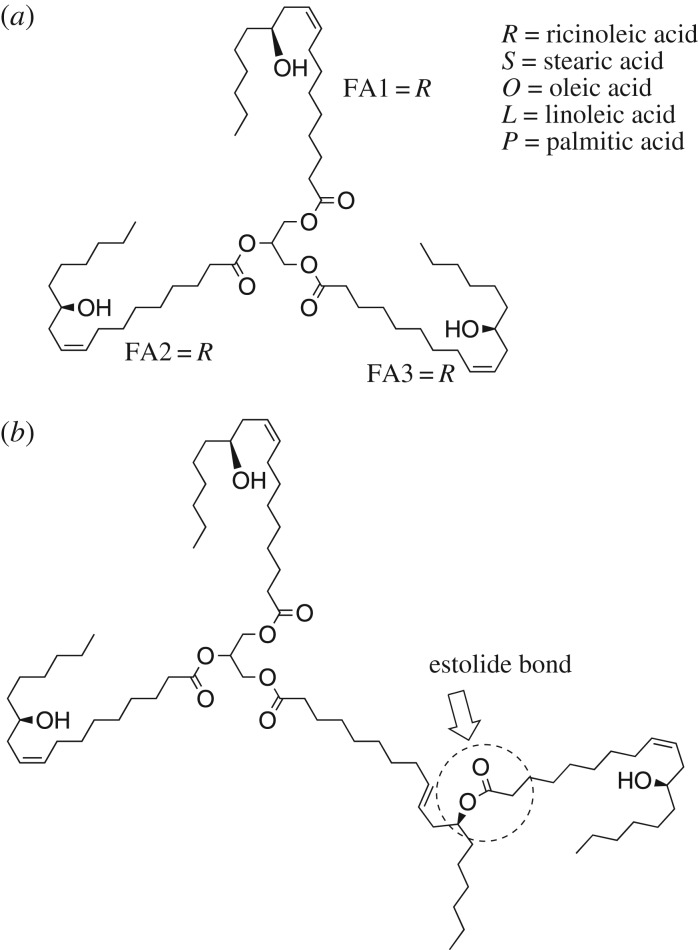
(*a*) Triglycerides found in CO (FA1, FA2 and FA3 represent FAs attached to the glycerol, when FA1 = R, FA2 = R, FA3 = R, i.e. triricinolein (RRR), diricinoleoystearoylglycerol (FA1 = FA2 = R, FA3 = S, i.e. RRS), diricino-leoyloleoylglycerol (FA1 = FA2 = R, FA3 = O, i.e. RRO), diricinoleoyllinoleoylglycerol (FA1 = FA2 = R, FA3 = L, i.e. RRL) and diricinoleoylpalmitoyl-glycerol (FA1 = FA2 = R, FA3 = P, i.e. RRP) and (*b*) estolide formation between a triglyceride molecule and a free FA (source: Isbell [48]).

Most of the triglyceride molecules in CO consist of three molecules of RA connected to a glycerol moiety [55]. Salimon *et al*. [27] identified five major triacylglycerides in CO, which are triricinolein (RRR) (84.1%), diricinoleoystearoylglycerol (RRS) (8.2%), diricino-leoyloleoylglycerol (RRO) (5.6%), diricinoleoyllinoleoylglycerol (RRL) (1.2%) and diricinoleoylpalmitoyl-glycerol (RRP) (0.9%). Table 3 gives the triglyceride composition of CO as reported by Ndiaye *et al*. [54]; Plante *et al*. [55] and Lin [47], however, reported the RRR content to be 63% and 70%, respectively. Lin [47] found four new diacylglycerols and eight new triacylglycerols in castor (figure 4). Again, Lin & Chen [56] found 40 new molecular species of acylglycerols in CO that are less polar than RRR (the most abundant triglyceride). The chain lengths of those acylglycerols were C16, C18, C20, C22 and C23. The number of double bonds ranged from 0 to 3, and the number of hydroxyl groups 0–3. Additionally, some estolides and tetraacylglycerols reported in CO are (12-ricinoleoylricinoleoyl)-ricinoleoyl-linoleoyl-glycerol (RRRL), (12-ricinoleoylricinoleoyl)-ricinoleoyl-oleoyl-glycerol (RRRO), (12-ricinoleoylricinoleoyl)-ricinoleoyl-palmitoyl-glycerol (RRRP), (12-ricinoleoylricinoleoyl)-ricinoleoyl-stearoyl-glycerol (RRRS) and (12-ricinoleoylricinoleoyl)-ricinoleoyl-linolenoyl-glycerol (RRRLn) [56]. These acylglycerols were analysed using high-performance liquid chromatography and electrospray ionization mass spectrometry, and the level of the total acylglycerols containing polyhydroxy FAs was only 3% of the CO, while the individual molecular species of acylglycerols containing polyhydroxy FAs were approximately 0.5% or less of CO [56]. Estolides are identified by the secondary ester linkage of one FA molecule to the alkyl backbone of another FA fragment. Estolides can be in the form of free acids, esters or could be found within the structure of triglycerides. Estolides usually form in most hydroxylated oils such as castor and Lesquerella oils [48].

**Table 3.** Triglyceride composition of CO [54].

triglyceride	% composition
triestearin	0.9
tripalmitin	1.4
triolein	3.5
trilinolein	4.9
trilinolenin	0.3
triricinolein	88.9

### 3.2. Fatty acids

The uniqueness of CO compared with other VOs lies in its FA composition. Numerous groups have reported on CO FA composition from different countries (table 4). The FAs found to be present in CO are RA, linoleic acid, oleic acid, stearic acid and linolenic acid. Approximately 84–90% of the FAs is RA and 10–16% consists of the other FAs (table 4) [22,27,28,57,58]. The FA compositions varied only slightly from different countries. Da Silva *et al*. [49] also showed the CO composition as 89.5% RA, 3.7% linoleic acid, 3.0% oleic acid, 1.6% palmitic acid, 0.9% stearic acid, 0.6% behenic acid, 0.4% linolenic acid and 0.3% arachinic acid.

**Table 4.** FA composition of CO from different countries.

FA	Percentage				
	Malaysia [27]	Iran [57]	Israel [22]	Brazil [58]	Pakistan [28]
ricinoleic	84.2	87.57	87.90	88.20	90.20
linoleic	7.3	4.29	4.95	4.90	4.40
oleic	5.5	5.16	4.87	3.80	2.80
palmitic	1.3	1.32	1.00	1.40	0.70
stearic	1.2	0.51	1.00	0.9	0.9
linolenic	0.5	1.12	0.25	0.3	0.2

**Figure 5.**
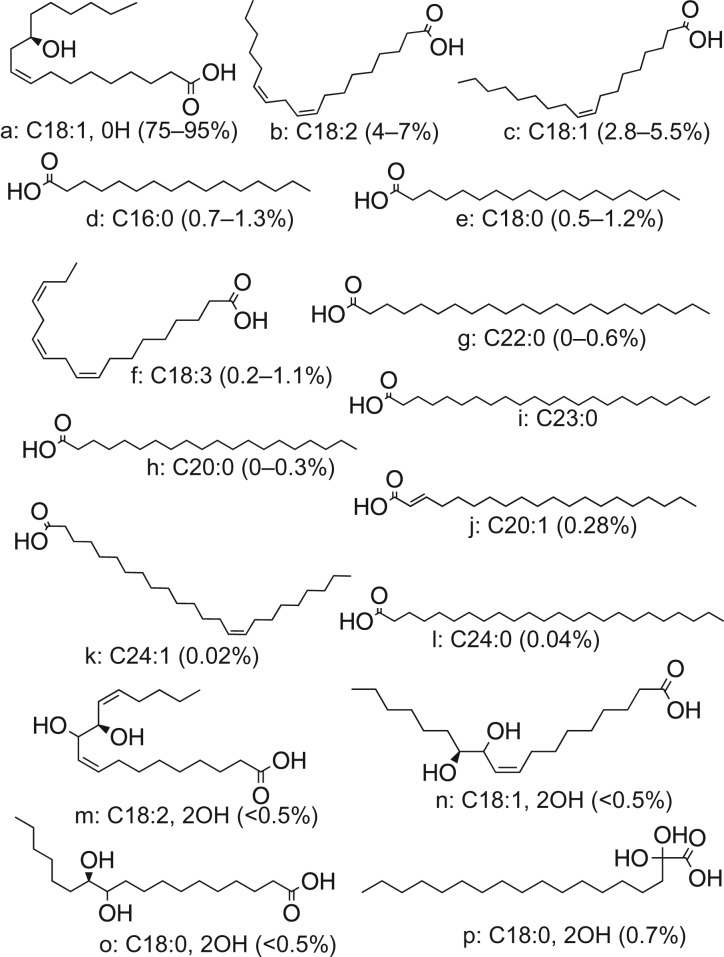
Chemical structures of FAs (percentage composition in brackets) found to be present in CO (a: RA, b: linoleic acid, c: oleic acid, d: palmitic acid, e: stearic acid, f: linolenic acid, g: behenic acid, h: arachinic acid (or eicosanoic acid), i: tricosanoic acid, j: eicosenoic acid, k: nervonic acid, l: lignoceric acid, m: 11,12-dihydroxy-9,13-octadecadienoic acid, n: 11,12-dihydroxy-9-octadecenoic acid, o: 11,12-dihydroxyoctadecanoic acid, p: dihydroxystearic acid) (source: Lin [47]).

Although CO is known to contain RA, which is a monohydroxy FA, Lin [47] has identified three new dihydroxy FAs in CO which they propose to be 11,12-dihydroxy-9-octadecenoic acid, 11,12-dihydroxy-9,13-octadecadienoic acid and 11,12-dihydroxyoctadecanoic acid (figure 5). Additionally, tricosanoic acid, which is an odd-numbered long FA (C23:0), has been identified to be present in CO by the same group [56]. Remarkably, Hosamani *et al*. [59] also reported *Alternanthera triandra*, Lam Syn. *Alternanthera sessilis* (L.) R. Br. seed oil as another source of RA (contains approx. 22.1% of RA). However, despite the possibility that other seed oils may contain RA, CO remains the only reported rich source of RA to date.

### 3.3. Isolation of ricinoleic acid from castor oil

Several methods, including chemical and biochemical pathways, have been used to isolate RA from CO. The isolation occurs by hydroxylation of the ester linkages in the triglyceride molecules to yield RA and glycerol. The salt solubility-based fractionation method reported by Vaisman *et al*. [22] is an example of a chemical method for isolating RA from CO. In this method, CO is hydrolysed by refluxing with an ethanol solution of KOH for 1 h, and the ethanol is evaporated to yield the potassium salt of the FA. The FAs are liberated by dissolving in deionized water and acidifying with concentrated HCl. The FAs are then extracted with ethyl acetate and dried over MgSO_4_. Clarification of the FAs is done by mixing with *n*-hexane (1 : 5 w/v) and keeping at −4°C for 72 h in darkness. Chromatographic analysis of the resultant FAs revealed the purity to be within 87.50–88.10% of RA and 12.5–11.9% of palmitic acid, stearic acid, oleic acid, vaccenic acid, linoleic acid and linolenic acid. Solid residues found after clarification were identified to be 9-,10-dihydroxystearic acid [22].

Biocatalysts such as lipase (triacylglycerol acylhydrolase, EC 3.1.1.3) enzymes have been used to isolate RA from CO. Foglia *et al*. [60] employed *Candida rugosa*, *Pseudomonas cepacia* and *Geotrichum candidum* lipases for hydrolysis of CO. In a typical reaction, tubes containing 100 mg of oil, 0.6 ml of 0.5 M phosphate buffer (pH 7) and approximately 2–5 mg of free lipase were stirred at 500 r.p.m. at 30°C for 1–4 h. The extent of hydrolysis was determined by titrating the hydrolysis mixture (in 20 ml of diethyl ether/ethanol/water (3 : 3 : 2)) to pH 12 with 0.1 N NaOH solution. The *P. cepacia* lipase was found to be effective in hydrolysing CO to RA to the tune of 27% compared with 13% recorded for *C. rugosa* and *G. candidum*. Ozcan & Sagiroglu [61] also employed immobilized *C. rugosa*, porcine pancreatic and castor bean lipases for lipolysis of CO and obtained a yield of RA within 20–40%, considering a number of parameters such as pH, temperature, amount of substrate and enzyme. Interestingly, Piazza & Farrell [18] used lipase from ground oats (*Avena sativa* L.) to hydrolyse CO and obtained approximately 90% yield of RA.

An eco-friendly approach by the use of microwave-assisted extraction of RA from CO has also been reported by Karpakavalli *et al*. [62]. In their approach, a 250 ml beaker containing 5 g of CO and a solution of ethanoic KOH with a few pieces of ice was covered and kept in a household microwave oven. The microwave oven was modified to contain a magnetic stirrer and a water condenser. Heating the reaction system continuously with the microwave oven at 160 W intensity for 19 min, 89% yield of RA was obtained. This microwave-assisted technique was found to be efficient compared to conventional heating of the hydrolysis system. All the above approaches for isolation of RA from CO show a good yield but differ only in the use of different catalysts and sources of heat.

## 4. Applications of castor oil

**Figure 6.**
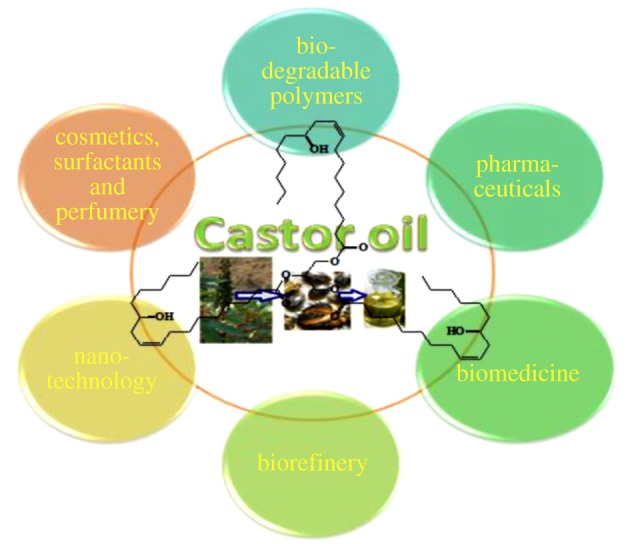
Application of CO in different fields.

CO has received much attention as a valuable commercial feedstock for production of a variety of products in a wide range of industries spanning pharmaceuticals to lubricants. Figure 6 shows a chart illustrating the different areas of CO application reported in the literature.

### 4.1. Biomedicine and pharmaceuticals

Historically, CO has been known as a medicinal oil and primarily used as a purgative or laxative to ease constipation [16]. As far back as 500 BC, the Egyptians used CO for purging purposes [40]. According to Anjani [40], an ancient Egyptian treatise, Ebers Papyrus, in 1552 BC later described CO as a purgative. CO is also a known cathartic agent used to induce labour in females. Tunaru *et al.* [17] found that CO induces laxation and uterus contraction as the RA released from the CO by intestinal lipases activates the prostaglandin EP_3_ receptors.

Additionally, eye drops containing approximately 1.25% of homogenized CO are reported for the treatment of lipid-deficiency dry eye (i.e. meibomian gland dysfunction). The role of CO in treating dry eye is that it serves as a hydrophilic lipid that spreads over the human tear aqueous layer to correct the deficiency [63].

Katzer *et al*. [64] have also reported that RA has some potential anti-inflammatory properties. Undecylenic acid (a chemical derived from CO) is also reported to be an antifungal and antiviral and has been used as a chemical building block for vital compounds that possess mosquito repellent, cytotoxic and antibiotic activity [38].

### 4.2. Polymer synthesis

The use of CO as a raw material in the synthesis of polymeric materials is very well established. The hydroxyl functionality is more suitable for isocyanate reactions, yielding polyurethane, while the double bond is dehydrated to obtain dehydrated CO, which is applied in producing paints, enamels, lacquers and varnishes [80]. Polymers of CO are applied in various fields such as wound dressing, drug delivery, bone tissue engineering and membranes for fuel cell fabrication. Yari *et al*. [81] reported on a novel antibacterial and cytocompatible polyurethane membrane based on CO for wound dressing. The synthesis of this polymer employed the hydroxyl functional groups on the CO molecule as the anchoring groups to hold the antibacterial agent. The RA-based polyanhydrides are also reported to possess the desired physico-chemical and mechanical properties for use as drug carriers, and *in vitro* studies showed that these biopolymers degrade rapidly via hydrolysis after 10 days, releasing RA and its counterparts [82]. Table 5 shows the role of CO in different applications or products.

**Table 5.** The role of CO in different applications or products.

description	application/product	role of CO	references
coatings	polyamidoamine toughened epoxy coating	curing agent	[65]
polyurethane	polyurethane films for protein adsorption	cross-linking agent	[66]
biofuels	biodiesel	triglyceride	[67]
membranes	polyurethane/polyaniline membranes for electrodialysis and fuel cells	polyol	[68]
polyurethane	microcellular polyurethane	polyol	[69]
interpenetrating polymer	hydrogenated CO uralkyd resin/poly(butyl acrylate) blend	polyol	[70]
methoxycinnamic oil	sunscreen active ingredient	hydroxy oil	[71]
polymer composite	dehydrated CO epoxy/poly(methacrylic acid) blend	additive	[72]
thermosetting resins	FA-based co-monomer for styrene replacement	plasticizer	[73]
hyper-branched polyester	polyester	methyl 10-undecenoate	[74]
polyester	CO-based organogels	methyl ricinoleate	[75]
biochemicals	γ-decalactone	ricinoleic acid	[76]
biosurfactants	sophorolipids	non-conventional carbon source/substrate	[77]
organic–inorganic hybrid films	epoxidized CO/-3-aminopropyltriethoxysilane	alkene	[78]
non-ionic surfactants	polyethylene oxide monomethyl ether-based CO	alkene	[79]

### 4.3. Cosmetics, perfumery, surfactants and biofuels

Very important industrial chemicals such as γ-decalactone, sophorolipids, undecylenic acid, linoleic acid, sebacic acid, capryl alcohol, heptaldehyde, zinc ricinoleate, glyceryl ricinoleate and lithium 12-hydroxystearate are produced from CO [76,77]. These chemicals have important roles in cosmetics, perfumery and surfactants, and even in polymer synthesis. Interestingly, Compton *et al*. [71] synthesized a novel sunscreen active ingredient, methoxycinnamic oil (MCO), using CO as the hydroxy oil. CO was reacted with 4-methoxycinnamic acid to yield MCO, which possessed broad UV absorbance from 250 to 345 nm, with the maximum at 305 nm. Another area where CO is massively used is in the production of biofuels. In 4000 BC, CO was being used as a fuel in wick lamps for lighting in ancient Egyptian tombs [43]. Biodiesel has been produced by transesterification of CO [67].

## 5. Syntheses and surface functionalization of nanoparticles

Green syntheses of nanoparticles are strongly advocated worldwide because of the disadvantages of the use of toxic solvents and chemicals, especially the effects on human health and the environment. Green chemistry principles embody the (i) design of less hazardous chemical syntheses, (ii) use of safer chemicals and solvents, (iii) use of renewable feedstocks and (iv) design of degradation [83]. Thus, renewable bioresource materials are currently the choice of raw materials for most nanochemistry researchers. VOs and FAs are used in green nanochemistry syntheses because they are:

(i) environmentally benign and inexpensive;
(ii) suitable alternatives to some toxic and expensive solvents or ligands traditionally used in nanoparticle syntheses;
(iii) renewable source of raw material;
(iv) biodegradable and provide versatile chemistry-based opportunities;
(v) a source of carboxylic acids suitable as ligands/capping agents or for synthesizing safe chemical precursors for metal oxide and sulfide nanoparticle syntheses; and
(vi) biocompatible, ensuring dispersion of nanoparticles in non-polar solvents.

For biomedical applications (e.g. staining of proteins), nanoparticles should be: (i) biocompatible, (ii) water soluble and (iii) easily functionalized or chemically modified at the surface to tailor the interaction of the nanoparticles with target biomolecules [30,83,84]. Fundamentally, ligands used for nanoparticle surface functionalization must: (i) have minimal cytotoxicity and (ii) be specific to the targeted biomolecule. VOs and FAs meet these requirements perfectly [30]. Thus, CO, olive oil, sunflower oil, almond oil, rapeseed oil, corn oil, palm oil and coconut oil have all been applied for syntheses of metal, metal chalcogenide and up-conversion of nanoparticles as well as biodegradable nanocomposites (table 6). Oleic acid and stearic acid have traditionally been massively employed in nanoparticle syntheses as both capping agents and solvents. RA has also recently received attention as a suitable alternative to oleic acid. CO together with RA has extra advantages that are not common to the advantages reported for edible oils:

(i) CO is inedible and obviates possible competition as raw material for the food industry;
(ii) CO is a natural source of polyol and presents a simple avenue for versatile chemical reactions;
(iii) CO is the only rich source of RA that has been used as a building block for synthesis of several biochemicals;
(iv) RA due to the presence of the hydroxyl functional group on its hydrocarbon chain provides a facile route for chemical functionalization and manipulation of nanoparticle surfaces to tailor it to a specific application;
(v) CO and RA are more suitable for applications requiring highly polar organic solvents; and
CO and RA possess antimicrobial properties.

**Table 6.** Different VOs and FAs employed in nanoparticle syntheses.

solvent/capping agent	nanoparticles	method	morphology	size (nm)	references
CO	CdS	colloidal thermolysis	spherical	4.64	[14]
CO	Ag	laser ablation	spherical	5	[86]
CO	Au	sputtering	spherical	2.4–3.8	[87]
CO	Au	wet chemical synthesis (i) NaBH_4_, (ii) citrate, (iii) KOH	quasi-spherical	(i) 9 (ii) 66 (iii) 13	[2]
*other VOs* (*edible*)					
rapeseed oil	Fe	sonochemical synthesis	spherical	20–30	[85]
corn oil	Fe	sonochemical synthesis	spherical	10–15	[85]
palm oil	Ag	laser ablation	spherical	2–2.5	[88]
coconut oil	Au	wet chemical synthesis	triangular to nearly spherical	38–49	[15]
coconut oil	Ag	wet chemical synthesis	triangular to nearly spherical	21	[15]
sunflower oil	ZnO	colloidal thermolysis	spheroidal	3	[89]
sunflower oil	Fe_2_O_3_/Fe_3_O_4_	colloidal thermolysis	spheroidal	7	[89]
almond oil	Mn_3_O_4_	sonochemical synthesis	spherical	7	[90]
olive oil	Mn_3_O_4_	sonochemical synthesis	spherical	7	[90]
olive oil	ZnS	colloidal thermolysis	dots to flower-like	4–7	[91]
olive oil	CdSe	colloidal thermolysis	dots	6	[92]
olive oil	PbS	colloidal thermolysis	cubic	18.74	[93]
olive oil	CdS	colloidal thermolysis	spherical	4.75	[93]
olive oil	Fe_2_O_3_/Fe_3_O_4_	co-precipitation	spherical	19.2	[94]
olive oil	CdSe	colloidal thermolysis	spherical	2.3–6	[95]
olive oil	PbS	colloidal thermolysis	—	3.4–4.7	[96]
*CO-based FA*					
RA	CdS	colloidal thermolysis	spherical	5.56	[14]
RA	Fe_3_O_4_	co-precipitation	Janus-type	—	[97]
RA	Fe_2_O_3_/Fe_3_O_4_	Co-precipitation	—	11.1	[98]
RA	CoFe_2_O_4_	co-precipitation	spherical	15	[99]
RA	NaYF_4_:Yb/Er	solvothermal	spherical	20	[100]
*other FAs*					
stearic acid	CdSe	colloidal thermolysis	dots	4–25	[32]
oleic acid	Fe_2_O_3_/Fe_3_O_4_	co-precipitation	—	10.4	[98]
oleic acid	PbS	chemical co-deposition	—	8	[101]

### 5.1. Metal nanoparticles

Metal nanoparticles are synthesized either by wet chemical, laser ablation, sputtering deposition or sonochemical methods. Diphenylmethane is a common solvent used in sonochemical reactions. Diphenylmethane is, however, reported to decompose to toxic by-products [85]. In addition, diphenylmethane is an expensive solvent and Koltypin *et al*. [85] have demonstrated that VOs are the best alternatives. Edible VOs such as rapeseed, corn, coconut and palm oils have been used as cheap and environmentally friendly solvents for the synthesis of silver, gold and iron nanoparticles (table 6) [2,15,85–88]. Thermodynamically, nanoparticles are bent on agglomeration to form larger particles. VOs and FAs are amphiphilic molecules that are used to effectively control nanoparticle agglomeration. The polar or hydrophilic end (carboxylic group) of the FAs interacts with the nanoparticles while the non-polar aliphatic tails or hydrophobic end disperses the particles by a steric effect [86]. However, the use of edible oils as solvents in an advancing nanotechnology industry presents an intrinsic problem that may relate to cost and competition for raw materials.

As previously stated, CO is inedible and the seed oil is high yielding compared with most edible oils, and is found to be the best and inexpensive alternative to edible oils for metal nanoparticle syntheses [87]. In addition, the high viscosity, high polarity, low vapour pressure and relative optical activity of CO makes it a more suitable stabilizer compared to other VOs [86,87]. In a sustainable approach, Zamiri *et al*. [86] combined laser ablation (considered a green method) and CO (as solvent) to provide a more sustainable green synthetic route for silver nanoparticles. Likewise, Da Silva *et al*. [2] made colloidal solutions of gold nanoparticles using CO as a non-toxic organic dispersant and/or a stabilizing agent and proved that CO nanoparticle colloids remain completely stable even after 3 months. The stability of the CO colloid was assumed to be a result of hydrogen bonding between the hydroxyl group on CO and the oxygenated negatively charged surface of the gold nanoparticle (produced via decomposition of HAuCl_4_ in KOH solution), because several attempts to synthesize similar stable colloids with soya bean or cottonseed oils failed [2].

Interestingly, antimicrobial paints based on VOs and silver nanoparticles have been developed via a simple method centred on free radicals generated *in situ* by autoxidation of the drying oil [102]. Drying oils (hardening on exposure to air) are preferred to non-drying oils in the making of paints [102]. However, CO is a non-drying oil, but can be dehydrated to obtain semi-drying or drying oil useful for developing such antimicrobial coatings [16].

### 5.2. Metal chalcogenide semiconductor nanoparticles

Metal chalcogenide semiconductor nanoparticles are a useful class of inorganic materials that have received tremendous applications in solar cells and biomedical labelling [103]. Some key chemicals used in the traditional syntheses of magnetic and luminescent nanoparticles (such as TOPO and TOP) are extremely toxic, pyrophoric, explosive and/or expensive, and therefore have a serious negative environmental impact [32]. In this regard, many phosphine-free synthetic methods have been proposed by different research groups to avoid the use of these toxic solvents. One common green route often reported is the use of VOs and FAs as coordinating solvents for synthesis of metal chalcogenide nanoparticles. Examples of VOs and FAs that have been used are olive oil, sunflower oil, almond oil, CO, oleic acid, stearic acid and RA [14,32,89–93,98,99]. Metal oxides, sulfides and selenides have been prepared using VOs and FAs as: (i) both solvent and capping agent; (ii) co-solvent (mixture of solvents); (iii) capping agent or ligand; and (iv) metal FA salts.

#### 5.2.1. Vegetable oil as both solvent and capping agent

Xiao *et al*. [91] demonstrated the shape evolution of ZnS nanoparticles in a green chemistry approach by employing olive oil as both solvent and capping agent. Specifically, a solution (made of 0.2 mmol of sulfur powder and 1 ml of olive oil, prepared at 100°C) was swiftly injected into a hot ZnO powder solution (consisting of 5 ml of olive oil and 0.4 mmol ZnO powder) at 330°C (Schlenk line with nitrogen gas). Dot- and flower-like morphologies of ZnS were obtained when there was sufficient ligand protection and limited ligand protection, respectively. Ligand protection is associated with the passivation of the surface of the nanoparticles by the ligand. Similarly, Bera *et al*. [104] prepared high-quality CdSe nanocrystals using olive oil. Their procedure consisted of three steps: (i) selenium powder (0.25 mM) was dissolved in 5 ml of olive oil at 220°C, (ii) CdO powder (0.5 mM) was dissolved in 25 ml of olive oil at 300°C, and (iii) the selenium solution was injected into the Cd, keeping the CdSe growth at 300°C. The TEM micrograph and electron diffraction pattern of the olive oil-capped CdSe showed that they were spherical and had an average size of 6 nm, zinc blende in nature. Mondal *et al*. [92] and Hardman *et al*. [96] replicated similar procedures to synthesize CdSe and PbS nanoparticles using olive oil.

Though olive oil was reported to be a suitable green solvent, Hardman *et al*. [96] noted that the hydrophobic groups were highly insulating and limited the as-prepared olive oil-capped nanoparticles for applications requiring charge transport to and from the nanoparticles. Thus, ligand-exchange processes are always required to tailor the nanoparticles to a particular application.

#### 5.2.2. Vegetable oils and fatty acids as co-solvent

Solvents such as octadecene and TOPO are often used as co-solvents with VOs and FAs. The reasons for the co-solvent addition are to: (i) decrease the viscosity of the oil to ensure uniform nucleation of the nanoparticles and (ii) reduce the strong binding of the FAs to the nanoparticles or reduce the extent of inhibition of the nanoparticle growth [29,105]. Akhtar *et al*. [105] synthesized high-quality PbS nanoparticles at 60°C in an olive oil/oleic acid/octadecene system (12.5 ml of olive oil, 1 ml of oleic acid and 1 ml of octadecene). The reasons stated for the co-solvent were for the oleic acid to dissolve the PbO and the octadecene to reduce the viscosity of the olive oil. The PbS nanoparticles were spherical and had sizes within 2.79–5.22 nm.

Nyamen *et al*. [93] thermolysed heterocyclic dithiocarbamate single-source precursors of Pb and Cd in an olive oil/TOPO solvent system and obtained an average size of 18.47 nm and 4.75 nm of PbS and CdS nanoparticles, respectively. The use of the single-source precursors makes the process greener and obviates problems related to stoichiometry. Qu *et al*. [32] synthesized high-quality wurtzite CdSe nanocrystals using stearic acid as the solvent and TOP or tributylphosphine as the injection solvent. The reason for adding the co-solvents (with relatively slow solidification rates compared to stearic acid) was to ensure ease in taking sample aliquots. Dickerson *et al*. [106] also, applying a similar system (5% of TOPO and 95% stearic acid), added TOPO as a co-solvent to ensure adequate coordination of ligands.

Qu *et al*. [32] revealed that FAs–co-solvent systems are: (i) versatile and more reproducible than TOPO alone; (ii) not recommended for synthesis of very small nanocrystals because of the fast growth rate of nanoparticles in such systems; and (iii) suitable for broad nanocrystals with a size distribution (2–25 nm), compared with purely phosphonic acid/TOPO systems that are more suited for synthesizing monodispersed (6–8 nm) strong confinement size regimes of nanoparticles. Dickerson *et al*. [106] found the activation energy of nanoparticle growth rate to be dependent on the MW of the solvent. The average activation energies were determined for CdSe nanocrystal growth in stearic acid (with MW of 284.47 g mol^−1^) and in TOPO (with molecular weight of 386.65 g mol^−1^) to be 0.56 and 0.95 eV molecule^−1^, respectively [106]. Thus, the addition of TOPO as a co-solvent helps to reduce the fast growth rate of nanoparticles in FAs, thereby producing high-quality nanoparticles. Though extensive work has been reported on the kinetic study of nanoparticle growth in FAs, less or no focus has been paid to kinetics related to the growth of nanoparticles in VOs (with molecular weights between 800 and 950 g mol^−1^, higher than the molecular weight of TOPO).

#### 5.2.3. Fatty acids as capping agents

**Figure 7.**
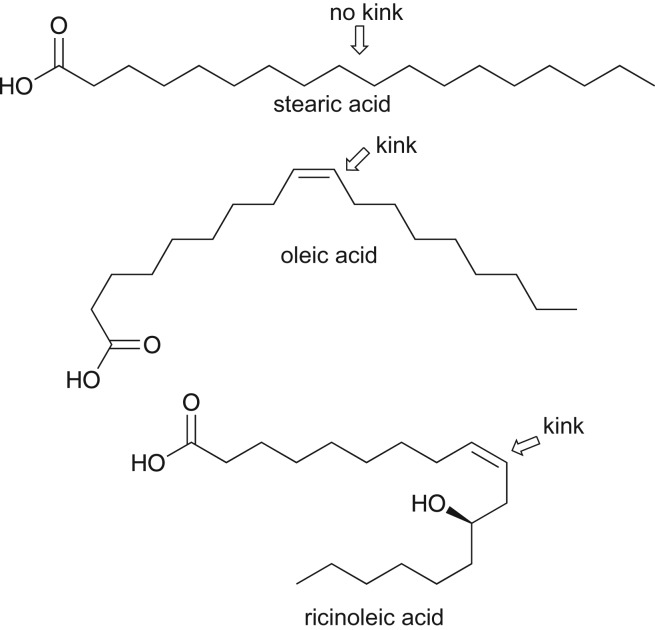
Chemical structures of stearic acid, oleic acid and ricinoleic acid. The kink in the structures is shown by an arrow [98].

FAs are Lewis acids and have been extensively applied as capping agents and surfactants in the syntheses of nanoparticles. Oleic acid is known as a standard FA; the double bond and alkyl chain forming a ‘kink’ imparts colloidal stability [107]. Oleic acid is found to be more efficient in stabilizing magnetic nanoparticles than stearic acid, which has a no ‘kink’ in its chemical structure (figure 7) [29]. A more closely related FA to oleic acid is RA. RA (derived from CO) is isostructural with oleic acid and has also received considerable attention for stabilizing and capping of magnetic and luminescent nanoparticles [14,97–99]. Table 7 gives the distinction between oleic acid and RA (related to their nanoparticle surface passivation, stabilization and application). One intriguing difference between RA and oleic acid is that the former has an OH in its carbon chain that is susceptible to reactions such as acetylation and polymerization, providing an avenue for small RA-capped nanoparticles to be coated with polymers for specific applications such as drug delivery. RA also provides an avenue for further chemical functionalization or modification of nanoparticles to improve on their dispersion or solubility in varying solvent media. In addition, for applications requiring highly polar organic solvents, RA is preferred over oleic acid. Unfortunately, due to the possibility of oxidation of the OH functional group to ketones, RA is less preferred than oleic acid in high-temperature organometallic synthesis of monodispersed nanoparticles [98]. However, oleic acid can be replaced with RA in a ligand-exchange process [107].

**Table 7.** Difference between oleic acid and ricinoleic acid [98].

oleic acid	ricinoleic acid
1. Is a C18 FA with one double bond between C9 and C10	Is isostructural with oleic acid but has a hydroxyl group at C12 in the C18 tail
2. The double bond in the middle of its carbon chain forms a ‘kink’ believed to be effective at nanoparticle stabilization	Has a similar ‘kink’ for nanoparticle stabilization. Provides a functional group (OH) in addition to the steric repulsion: (i) the OH can be acetylated under mild conditions, (ii) the OH has low affinity for iron oxide surfaces, and (iii) the OH could initiate ring opening polymerization reactions
3. Only colloidal suspensions in non-polar organic media can be prepared (oleic acid-coated nanoparticles cannot be dispersed in organic media with a dielectric constant larger than 5)	Colloidal suspensions in more polar organic media can be prepared
4. Suitable for capping of monodispersed nanoparticles synthesized at very high temperatures	Not suitable for high-temperature synthesis due to possible oxidation of the OH groups to ketones

#### 5.2.4. Metal fatty acid salt

Metal FA salts (MFASs) are polyvalent metal soaps, prepared by: (i) metathesis of a sodium or potassium FA salt with metal salts in aqueous or polar solvents, (ii) dissolution or fusion of metal oxides (or hydroxides, oxy-hydroxides, hydrocarbonates and carbonates) in hot FAs, or (iii) direct reaction of metal with hot FAs [31]. MFASs have become attractive as precursors for large-scale synthesis of metal oxide and metal chalcogenide nanoparticles because they: (i) are environmentally benign and (ii) yield reproducible results. Pereira *et al*. [89] combined MFAS (iron and zinc oleates) single-source precursors and sunflower oil (as solvent) for the synthesis of iron oxide and zinc oxide nanoparticles (at 200–250°C). Iron oxide and zinc oxide nanoparticles with average diameters of 7 and 3 nm and spheroidal in shape were obtained. While this route is considered green, the sunflower oil was prone to autoxidation via the double bonds in the oil at elevated temperatures (such as 310°C). Decomposition products such as ketones, esters, aldehydes, carbonates and carboxylic acids were identified. Factors such as temperature, UV light and metal ion complexes (iron and tin) were suggested to have accelerated the autoxidation process. Thus, choosing the right organic solvent (with a suitable boiling point) to decompose MFASs is crucial to obtaining monodispersed nanoparticles.

Chen *et al*. [31] decomposed an iron–oleate complex in five different organic solvents (oleylalcohol, benzyl ether, octadecene or trioctylamine)/oleic acid mixtures. Typically, the MFASs and the solvent mixture were placed in a flask and refluxed at the boiling point of the respective organic solvent for an hour to decompose the precursor. The outcome was monodispersed spherical iron oxide nanoparticles with sizes between 4.5 and 20.4 nm. The sizes were found to be dependent on both the boiling point of the organic solvent and the amount of oleic acid. The oleic acid was found to control the decomposition of the MFASs and the growth of the nanoparticles. By contrast, Cha *et al*. [108] decomposed iron–oleate complexes in the absence of an organic solvent (i.e. a solvent-less method) and obtained similar monodispersed iron oxide nanoparticles. By varying the annealing time and vacuum pressure, different shapes (spherical, regular triangular, short rod, diamond and long rod shapes) of iron oxide were produced with a mean size of 10.6 nm. The decomposition of MFASs follows equations (5.1) and (5.2):

5.1 $$\text{M}-\text{OOCR}\to {\text{M}}^{∙}{+}^{∙}\text{OOCR}$$

$$\text{M}-\text{OOCR}\to \text{M}{\text{O}}^{∙}+\text{O}{\text{C}}^{∙}\text{R}$$

MFASs decompose thermally through the formation of free radicals that combine, disintegrate into smaller molecules or react with other metal carboxylates to propagate to decompose metal carboxylates in MFASs [108].

Choi *et al*. [109] synthesized Cu_2_S, MnS, PbS, CdS and ZnS nanocrystals by the solution-phase thermolysis of metal–oleate complexes in alkane thiol. This method was considered simple and general for the synthesis of metal chalcogenides. Specifically, the metal–oleate precursors were dissolved in solvent mixtures of oleylamine and dodecanethiol. The resultant mixtures were then heated to the required temperatures and maintained for a period. The reaction temperature, time and the molar ratio of the two solvents were varied to tune the sizes of the nanoparticles. The nanoparticle sizes were uniform and had average particle sizes of 18, 11, 47, 10 and 10 nm for Cu_2_S, MnS, PbS, CdS and ZnS nanocrystals, respectively. Similarly, Patel *et al*. [110] obtained uniform-sized CdS, ZnS and PbS nanoparticles by sulfurization of metal–oleate precursors with thioacetamide at 140°C. Thermogravimetric and infrared studies indicated that the FAs were strongly coordinated or attached (symmetrically) to the surface of the nanocrystals via the carboxylate functional group. Metal–oleate complexes have been extensively explored for synthesis of metal chalcogenide nanocrystals. However, metal–ricinoleate complexes (prepared by reacting RA with metal salts) have not been explored, though RA is isostructural with oleic acid.

### 5.3. Up-conversion nanoparticles

**Figure 8.**
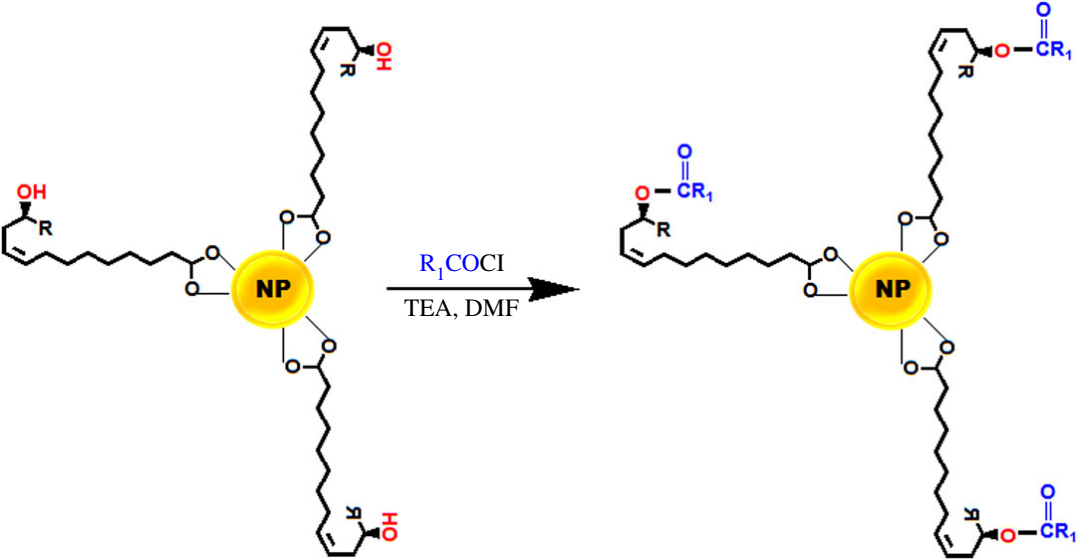
Reaction scheme showing the surface functionalization of ricinoleic acid-capped nanoparticles with dodecanoyl chloride. R = CH_3_(CH_2_)_5_, R_1_ = CH_3_(CH_2_)_10_ (adapted from He *et al*. [100]).

Up-conversion nanoparticles (UCNps) have received considerable attention as fluorophores in bioimaging over organic fluorophores and semiconductor quantum dots. UCNps have high quantum yields, high photostability and narrow emission peaks. However, to efficiently use UCNps in bioimaging, the UCNps must be rendered water-dispersible and their surfaces functionalized [100]. To render UCNps water-dispersible, hydrophobic UCNps are first prepared using oleic acid (or oleylamine) and then a ligand exchange, ligand attraction, silica coating, ligand oxidation or epoxidation process is performed. These surface modification routes are limited due to (i) the inability of the chemical reagents to get to the double bond in the oleic acid (as a surface ligand) because of high steric hindrance, (ii) complexity of the phase-transfer processes, (iii) long reaction time, and (iv) increase in the mean size of nanoparticles after ligand exchange [100,111]. In an attempt to overcome these shortfalls, RA is suggested by Meesaragandla *et al*. [111] as an excellent ligand alternative to oleic acid in making nanocrystals water-dispersible because the hydroxyl group (at C12) in the RA facilitates diffusion of chemical reagents for hydroxylation of the nearby double bond (between C9 and C10). To demonstrate the reactivity of the hydroxyl groups, He *et al*. [100] reacted dodecanoyl chloride with RA-capped NaYF_4_:Yb/Er and obtained NaYF_4_:Yb/Er-C_12_ (figure 8). Interestingly, RA-capped UCNps retained their average size and shape and even exhibited strong up-conversion properties in different protic and aprotic solvents after hydroxylation of the double bond [111]. However, there are only a few studies reported on RA-capped UCNps.

### 5.4. Nanocomposite materials

The coating of surfaces of very small size monodispersed nanoparticles with polymers for specific application in biomedicine is a major challenge. Nanoparticle surfaces can be polymerized in various solvents with the appropriate polymer for a specific purpose if ligands on the surfaces are suitable for such polymerization reactions. RA (and CO) in this regard stands out as the most suitable ligand (because of its freely available hydroxyl functional group) compared to oleic acid. In an attempt to develop polymer-coated monodispersed Fe_3_O_4_ nanoparticles, Lattuada & Hatton [107] first (i) performed a ligand exchange to replace oleic acid on the surface of the nanoparticles with RA and then (ii) exploited the hydroxyl group (on RA) to initiate ring opening polymerization of polylactic acid, which grew on the surface of the nanoparticles. This facile flexible method of functionalization is suggested to be efficient in tailoring nanoparticle solubility in a variety of solvents for different useful applications.

**Figure 9.**
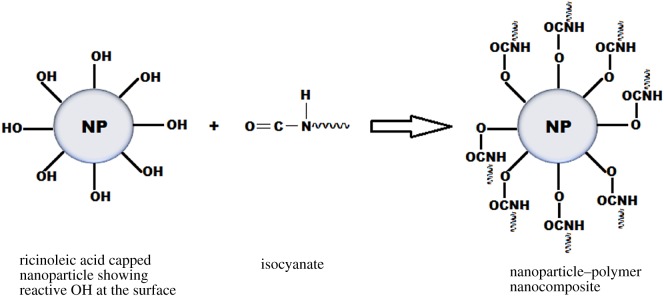
Isocyanate reaction with hydroxyl groups on RA-capped nanoparticle to form a nanoparticle–polymer nanocomposite [112].

Similarly, RA stabilized Fe_3_O_4_ nanoparticles composited with poly(lactic-co-glycolic) acid was reported by Furlan *et al*. [97] as a magnetically responsive drug delivery system. The Fe_3_O_4_ nanoparticles were synthesized using the Massart co-precipitation method. The RA played its role as a biocompatible ligand rendering the nanoparticles hydrophobic, which ensured their dispersibility in apolar and mildly polar organic solvents such as dichloromethane. Additionally, a wound healing bio-nanocomposite based on CO and chitosan-modified ZnO nanoparticles has also been reported [112]. The CO was used as the matrix material. The composites were made by mixing CO, chitosan-modified ZnO and hexamethylene diisocyanate in the presence of stannous octanoate as catalyst and glutaraldehyde as the cross-linking agent. The CO served as a polyol that reacted with the diisocyanate to form the polymer composite (figure 9). The polymer composites were said to be biocompatible and biodegradable. Xia & Larock [113] also prepared CO-based polyurethane (PU)–silica nanoparticle nanocomposites with an increased cross-link density that resulted in improved thermal stability and mechanical properties of the composite. The CO–PU was identified to be chemically bonded to the silica nanoparticles. The interface interaction in such organic–inorganic composites determines the properties of the composite. Thus, the availability of reactive OH groups on CO and RA guaranteed the strong chemical interaction between the composite materials.

## 6. Conclusion

CO has a useful versatile chemistry and has been reviewed as a valuable bioresource material for green syntheses of nanoparticles. It is used as a biocompatible solvent, co-solvent, and a capping and stabilizing agent in the syntheses of metal and metal chalcogenide nanoparticles, as well as a source of polyol for forming chemically bonded polymer–nanoparticle composites that are biodegradable. CO is distinct from other VOs and contains huge amounts of ricinoleic acid, which is isostructural to the traditional oleic acid for capping magnetic and luminescent nanocrystals [14,16,97,98,100,112].

Ricinoleic acid is a more suitable stabilizing and/or capping agent for applications requiring more polar organic solvents (e.g. in lubricants) [98]. It provides a facile route for surface functionalization of nanoparticles with different functional molecules to tailor their dispersion/solubility in different solvent media [107]. Functionalization is extremely necessary for various important applications, such as drug delivery, where nanoparticles are used. The free reactive hydroxyl group on ricinoleic acid enhances chemical interaction with different molecular species (such as polymers, isocyanates, acids and dodecanoyl chloride) [112]. Additionally, ricinoleic acid is a natural source of carboxylic acid and alcohol, and could be exploited for synthesis of (single-source) precursors for preparation of metal oxide and chalcogenide nanoparticles.

Though the utilization of CO and ricinoleic acid as solvent/capping/stabilizing agents for nanoparticle syntheses has substantial potential in expanding the spectrum of nanoparticle applications, it is somewhat limited. Ricinoleic acid is more suited for low-temperature organometallic synthesis because of the possible oxidation of the hydroxyl group on its hydrocarbon chain [98]. However, the various ways (shown in this review) that ricinoleic acid and CO can be used in nanoparticle syntheses have not been thoroughly explored.

## Data accessibility

This article has no additional data.
